# Revealing the WEDM Process Parameters for the Machining of Pure and Heat-Treated Titanium (Ti-6Al-4V) Alloy

**DOI:** 10.3390/ma14092292

**Published:** 2021-04-28

**Authors:** Nitin Kumar Gupta, Nalin Somani, Chander Prakash, Ranjit Singh, Arminder Singh Walia, Sunpreet Singh, Catalin Iulian Pruncu

**Affiliations:** 1 Department of Mechanical Engineering, DIT University, Dehradun 248009, India; ghotnitin@gmail.com (N.K.G.); nalin.somani41@gmail.com (N.S.); 2 School of Mechanical Engineering, Lovely Professional University, Phagwara 144411, India; 3 Department of Mechanical Engineering, National Institute of Technology, Jalandhar 144011, India; ranjitsingh.tmk@gmail.com; 4 Department of Mechanical Engineering, Thapar Polytechnic College, Patiala 147004, India; arminder.walia@gmail.com; 5 Department of Mechanical Engineering, National University of Singapore, Singapore 119077, Singapore; snprt.singh@gmail.com; 6 Department of Mechanical Engineering, Imperial College of London, Exhibition Road, London SW72AZ, UK; 7 Department of Design, Manufacturing & Engineering Management, University of Strathclyde, Glasgow G1 1XJ, UK

**Keywords:** WEDM, titanium alloy, material removal rate, surface roughness, cutting speed

## Abstract

Ti-6Al-4V is an alloy that has a high strength-to-weight ratio. It is known as an alpha-beta titanium alloy with excellent corrosion resistance. This alloy has a wide range of applications, e.g., in the aerospace and biomedical industries. Examples of alpha stabilizers are aluminum, oxygen, nitrogen, and carbon, which are added to titanium. Examples of beta stabilizers are titanium–iron, titanium–chromium, and titanium–manganese. Despite the exceptional properties, the processing of this titanium alloy is challenging when using conventional methods as it is quite a hard and tough material. Nonconventional methods are required to create intricate and complex geometries, which are difficult with the traditional methods. The present study focused on machining Ti-6Al-4V using wire electrical discharge machining (WEDM) and conducting numerous experiments to establish the machining parameters. The optimal setting of the machining parameters was predicted using a multiresponse optimization technique. Experiments were planned using the response surface methodology (RSM) technique and analysis of variance (ANOVA) was used to determine the significance and contribution of the input parameters to changes in the output characteristics (cutting speed and surface roughness). The cutting speed obtained during the processing of the annealed titanium alloy using WEDM was quite large as compared to the cutting speed obtained in the case of processing the pure, quenched, and hardened titanium alloys using WEDM. The maximum cutting speed obtained while processing the annealed titanium alloy was 1.75 mm/min.

## 1. Introduction

Excellent mechanical properties (hardness, fatigue resistance, tensile strength, toughness, etc.), high wear and corrosion resistance, good weldability, and a high strength-to-weight ratio are some of the major properties of titanium that make it suitable for making different dies, parts, molds, cavities, etc. [1]. Different reinforcements are added to the base material to improve the physical and mechanical properties of the material, which makes the material more suitable for different applications [2]. Titanium and its alloys have wide applications in different fields, such as in the marine engineering, aerospace engineering, petroleum refining, and chemical industries. Titanium alloys are used in a number of other industrial applications, including emission control, flue gas desulfurization, purified terepthalic acid (PTA) plants for polyester processing, pressure vessels, heat exchangers, and hydrometallurgical autoclaves. Each grade is customized to particular working conditions, with an emphasis on strength for various stresses, alloy quality for various corrosive agents, and ductility for various fabrication requirements [1,2,3,4,5]. Ti-6Al-4V (6% aluminum and 4% vanadium) is a titanium-based alloy that is widely used because of its high fatigue resistance and good corrosion resistance capacity. Table 1 shows the different properties of the Ti-6Al-4V alloy.

**Figure 1:**
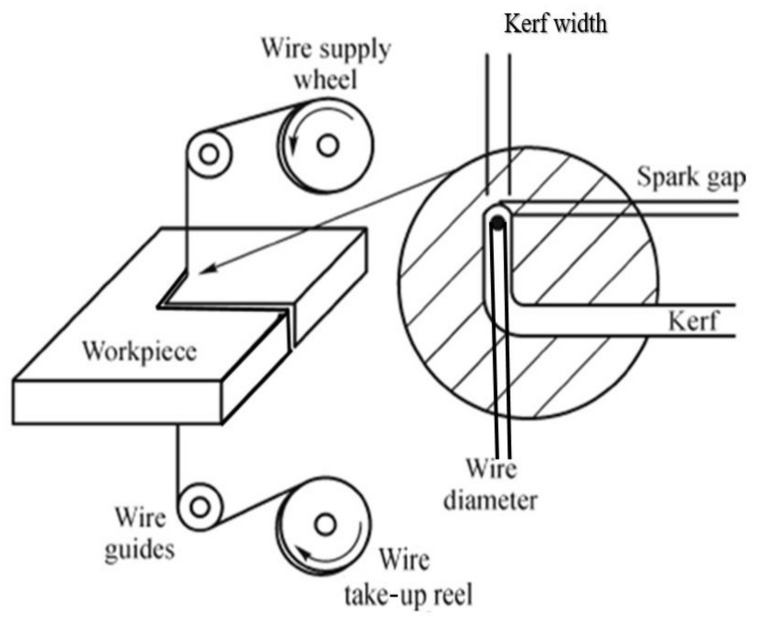
Wire electric discharge machining process [2].

Due to its high chemical reactivity (leading to tool deformation) and low thermal conductivity [7], titanium and its alloys are complicated to machine/process and not economical when used in traditional machining methods, such as reaming, grinding, milling, and turning. To work with such high-strength materials, wire electric discharge machining (WEDM) is widely used in many industries, which can machine with high precision and produce a good quality surface finish. WEDM is an advanced machining process that utilizes thermomechanical energy to remove materials during the cutting operation. During the WEDM process, a conductive wire that moves continuously acts as an electrode. The thermal energy is utilized during the machining and due to that electric spark, erosion of the conductive work material takes place. Dielectric fluid is used during the operation, which acts as an ionization medium and generates electrical sparks between the workpiece and the cutting wire. These electrical sparks develop thermal energy that removes material in the form of microdebris, while the continuous supply of dielectric fluid helps to flush away the removed material and acts as a coolant. Figure 1 shows a schematic diagram of the WEDM cutting principle.

For machining, the identification of suitable machining parameters is important for higher productivity and effectiveness. Achieving this optimization plays a very important role. The response surface methodology is one the most widely used optimization techniques that help to identify the most appropriate process parameters. In the present paper, the effect of different process parameters on the cutting speed while processing different heat-treated titanium alloys is discussed regarding the properties of the alloy for industrial and commercial applications. In this study, the response surface method was used for the design of the experimentation. The servo reference voltage (*V_sr_*), wire feed (*F_w_*), and wire tension (*T_w_*) were used as input process parameters, and the cutting speed was the output process parameter for the pure, annealed, hardened, and oil-quenched samples.

To improve the WEDM process performance, several studies have been carried out on titanium and its alloys so that better results, such as lower surface roughness and higher material removal rate, can be obtained. Shajan et al. [8] used Ti-6Al-4V as a work material and aimed to analyze the metallurgical changes of the work material. A 0.25 mm diameter brass wire with a zinc coating was used as the electrode wire. An L18 orthogonal array was used to design the experiment. Pulse duration, wire speed, servo voltage, wire tension, and pulse current were taken as the machining parameters. The study revealed that the pulse speed, wire tension, and wire speed were the most influential process parameters. Vamsi et al. [9] also worked on titanium-based alloys. An L27 orthogonal array was used to design the experiments and linear regression analysis was undertaken to create a mathematical model. The results showed that a better surface finish was obtained with a lower spark time and pulse current. Klocke et al. [10] undertook a comparative analysis between the grinding and WEDM processes for the machining of the Ti-6Al-4V alloy. Based on the results of the surface finish and visual inspection, they concluded that WEDM is better than the grinding process for machining titanium alloys. Basil et al. [11] used Ti-6Al-4V as a work material, where a brass wire of 0.25 mm diameter was used as an electrode. A two-level factorial method was used to model and predict the surface roughness. The input voltage, pulse-on time, dielectric fluid pressure, and pulse-off time were used as process parameters. The results showed that a better surface finish was obtained for a lower pulse-on time and at a lower dielectric fluid pressure. Alis et al. [12] investigated Ti-6Al-4V as a work material. Brass wire was used for machining a titanium alloy with a constant 4 A current. The discharge current, wire tension, and wire speed were selected as process parameters. Based on their study, they reported that a higher material removal rate was obtained at a higher current and a smooth surface was obtained by increasing the wire tension. It was also observed that the wire vibrations were reduced at a higher wire tension. Gupta et al. [13] used the WEDM process for machining a Ti-6Al-4V alloy with a constant 6 A current and with a varying machining rate from 2–6 mm/min at intervals of 2 mm/min. The material removal rate and surface roughness were the output parameters, while the pulse duration, wire speed, servo voltage, wire tension, pulse current, and feed rate were used as the process parameters. The best results were found using a servo voltage of 60 V, a wire tension of 1.4 kg, a speed of 8 m/min, and a feed rate of 4 mm/min. The surface quality was good at a lower machining feed rate and a higher wire tension. Some of the process parameters, such as pulse duration and wire speed, were noted as being less effective for material removal rate (MRR). For the machining of Ti-6Al-4V, Ghodsiyeh et al. [14] used a brass wire with a diameter of 0.25 mm. The design of experiments method was used to find the best process parameters. Deionized water was used as the dielectric fluid. As input parameters, they chose the pulse-on time, pulse-off time, and peak current. MRR and surface roughness (SR) were reported as the output process parameters. During the experimentation, they found that the peak current was the most influential process parameter, followed by the pulse-on time and pulse-off time. Kumar et al. [15] used the response surface methodology to model the parameters for the WEDM process on pure titanium. A Box-Behnken design was used to optimize the MRR, surface roughness, and electrode wear ratio. The machining parameters were the pulse-on time, pulse-off time, peak current, spark gap voltage, wire feed, and wire tension. The material removal rate increased with an increase in the peak current and a decrease in the pulse-off time. Nourbaksh et al. [16] used the Taguchi method for the optimization of the process parameters. An L18 orthogonal array was used for designing the experiments. The pulse current, servo voltage, wire current, and pulse width were selected as process parameters to measure the wire deformation, cutting speed, and surface integrity. Deionized water was used during the operation as the dielectric fluid. The results show that with an increase in the pulse width, the surface roughness increased. The servo voltage and wire current were noted as being less effective process parameters. For the machining of Ti-6Al-4V alloy using the WEDM method, Prasad et al. [17] used the pulse-on time, pulse-off time, servo voltage, and peak current as the machining parameters. The performance parameters were the rate of material removal and the roughness of the surface. To design the experiment and describe the significant process parameters, the Taguchi and ANOVA techniques were used. The peak current and pulse-on time were found to have the greatest impact on the material removal rate and surface roughness, while the pulse-off time and servo voltage had the least impact. Dhobe et al. [18] studied the WEDM mechanism and the impact of various heat treatment processes on machining using the pulse-on time, pulse-off time, peak current, and gap voltage as input process parameters.

The impact of the process variables on the surface roughness was investigated. For plain D2 steel and hardened D2 steel, an increase in the pulse-on time and a decrease in the pulse-off time were found to be beneficial for achieving a better surface finish. They also discovered that double tempering improved the surface finish as compared to single tempering. Mouralova et al. [19] used the WEDM process for machining Ti-6Al-4V alloy. The discharge current, gap voltage, pulse-on time, wire speed, and pulse-off time were taken as the process parameters to assess the surface integrity. During the experimentation, they found that a lower pulse-on time and a higher pulse-off time produced a better surface finish. The gap voltage was found to be a less influential process parameter. Pramanik et al. [20] investigated Ti-6Al-4V as a work material. The WEDM process was used for machining the titanium alloy. The pulse-on time, pulse-off time, and wire tension were used as process parameters. The surface roughness, crack propagation, and fatigue life were noted. Better results were obtained with a lower pulse-on time and a higher pulse-off time. In the previous research studies L9 design of experiment technique has been used to optimize self-healing materials [21,22,23].

A thorough literature search showed that many researchers around the globe have contributed in the area of process parameter optimization of the WEDM process regarding the machining of titanium-based alloys. Very limited literature was found regarding the WEDM of heat-treated titanium alloys. The titanium alloy was heat-treated at different temperatures (hardening, quenching, and annealing) and its machining properties were explored using the WEDM process. The machining characteristics of titanium alloy were investigated according to their wide range of applications in the industrial and commercial sectors. Unfortunately, there is little research that has discussed the effect of hardening on the machining performance of WEDM and no one has reported a comparative study on the optimization of process parameters of wire electro discharge machining on the different heat-treated titanium alloys. Therefore, systematic research was directed toward machining hardened titanium alloys and a comparative study of heat-treated titanium alloys.

## 2. Materials and Method

### 2.1. Materials

The Ti-6Al-4V and Cu wire were procured from the local Indian market (CDH, New Delhi, Delhi, India). Further Cu wire was used as the electrode tools in the WEDM as copper is a good conductor of electricity [24,25,26,27]. The diameter of the wire was 0.25 mm. The composition of the various component in the titanium alloy (Ti-6Al-4V) is tabulated in Table 2 (“pure titanium” is used for materials in the form it was received in from the market with the name Ti-6Al-4V).

### 2.2. Experimental Setup

**Figure 2:**
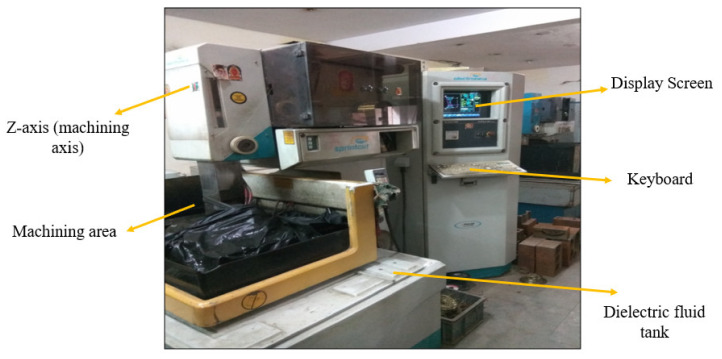
Pictorial view of the WEDM tool (courtesy: DIT University, Dehradun).

Figure 2 represents the actual machining setup of the wire EDM. The experiments were carried out on a five-axis numerical computer-controlled wire-cut EDM machine (EUROCUT MARK-2, Electronica Machine Tools Ltd., Pune, Maharashtra, India). The power supply unit, dielectric fluid tank, and machining tool are the main components of WEDM. The work material was connected to the positive terminal and the Cu wire electrode was connected to the negative terminal. The maximum travel capacity of the machine was 350 (X) × 400 (Y) × 250 mm (Z).

### 2.3. Methodology

**Figure 3:**
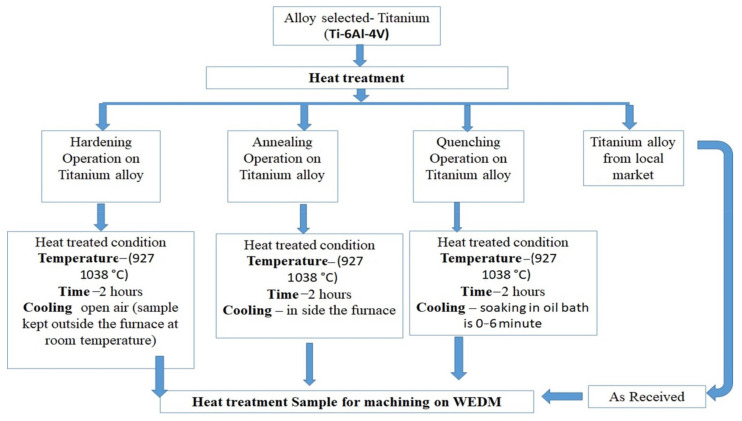
Methodology of the research.

Figure 3 shows the methodology of the research work, where various heat-treatment conditions were created for the samples. Heat-treatment processes involve heating and cooling a metal or alloy to alter their mechanical and physical properties to make them more desirable. The samples were treated as per the defined heat treatment conditions, followed by the wire electrodischarge machining process. Figure 3 summarizes the evaluation methodologies.

### 2.4. Selection of Process Parameters

Machining parameters, such as the servo reference voltage, wire feed, and wire tension, have a significant impact on the quality of the product generated by the WEDM process [28]. The right parameters help to produce a faster material removal rate and a smoother surface. The various input process parameters that were chosen for this experimental study, with their symbols, units, and value ranges are presented in Table 3. These parameters were selected based on a literature review [17,18,19,20,28] and pilot experimentations. The cutting speed was selected as the output process parameter.

### 2.5. Design of the Experiment

For the design of this experiment, the response surface methodology (RSM) technique was chosen. The RSM is a mathematical technique that is used for the optimization of process parameters. A Box-Behenken design was chosen for designing the matrix of experiments, as it leads to a greater number of experiments such that our experimentation data become more precise and accurate. In order to maintain the accuracy, each experiment was repeated three times. Experimentation was carried out to investigate the effect of the process parameters on the cutting speed. Table 4 represents the experimental design as per the RSM.

## 3. Results and Discussion

**Figure 4:**
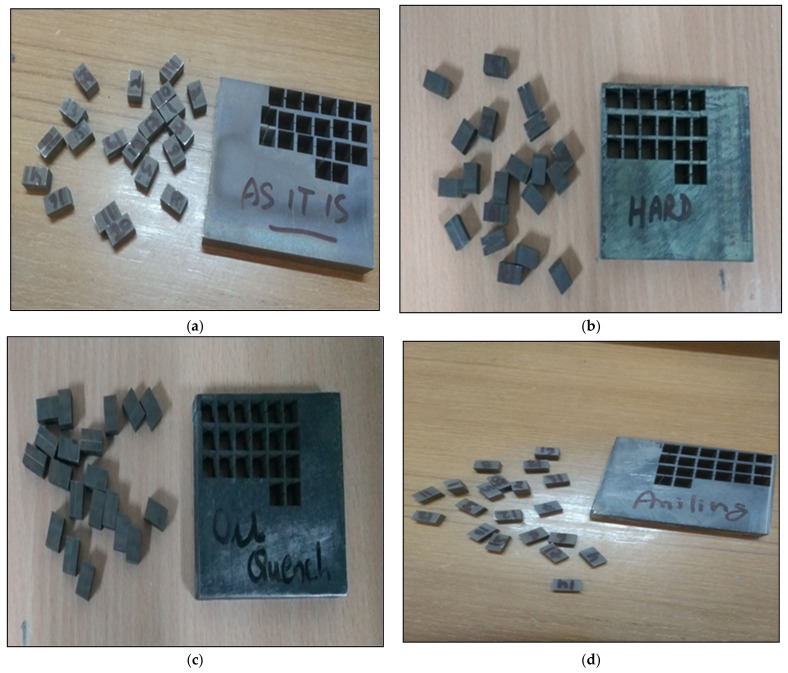
Actual samples cut using the wire EDM process. (**a**) Heat Treated Titanium Alloy as Received, (**b**) Hardening Heat treated Titanium Alloy, (**c**) Quenching Heat treated Titanium Alloy, (**d**) Annealing Heat treated Titanium Alloy.

Experimentation was performed on the titanium alloy under various heat-treated conditions and cutting was done as per the defined process parameters. Figure 4a–d shows the actual cutting sample that was created using WEDM. The samples that were processed were 15 mm × 10 mm × 10 mm rectangular bars.

The impact of the process parameters, such as servo reference voltage, wire feed, and wire tension, on one of the most critical response parameters, namely, cutting speed, was studied; the respective values were determined and tabulated in Table 4. The analysis for the machining of the titanium alloys was studied with the use of expert design software (DOE, V 13, StatEase, Minneapolis, MN, USA) and 3D graphs were analyzed to study the behavior of the cutting speed in response to the variations in the process parameters. Regression analysis was used to analyze the effects of the process parameters on the cutting speed. The Pearson correlation coefficient was 0.991, which shows a strong agreement of the prediction of the built model with the results of the experiment. The machined surface morphology was studied using FE-SEM (JSM-IT800, JEOL, New Delhi, India). The micrographs revealed the topography of the surface of the various processed titanium alloys.

### 3.1. Analysis of the Pure Titanium Sample

**Figure 5:**
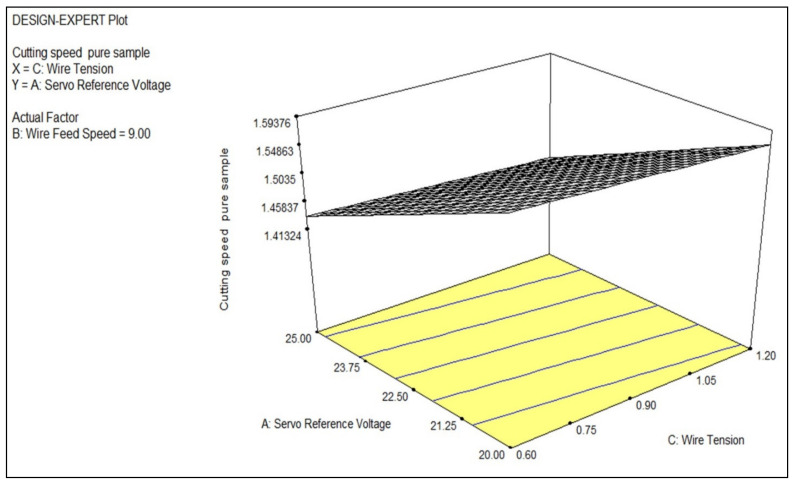
A 3D plot for studying the effect of the servo reference voltage and wire tension on the cutting speed.

The Figure 5 shows how the cutting speed changed as the servo reference voltage and wire tension changed. The increased wire tension tightened the wire, resulting in less slack and more sparks forming, which contributed to an increased cutting speed. The cutting speed increased as the servo reference voltage increased since a higher voltage results in further ionization of the dielectric fluid, which contributed to a higher discharge energy per spark. The cutting speed was increased as a result of the high discharge energy. As the pure titanium alloy was processed using WEDM, the combined effect of both the wire tension and servo reference voltage resulted in an improvement in cutting speed.

The following terms were used in the equations: servo reference voltage (*V_sr_*), wire feed (*F_w_*), and wire tension (*T_w_*).

The equation for the cutting speed when machining the pure titanium sample was as follows:

(1) $$Cutting\;Speed\;\left(Pure\right)=+2.13215-0.031765\times V_{sr}+0.013180\times F_{w}-0.036167\times T_{w}$$

### 3.2. Analysis of the Annealed Titanium Alloy

**Figure 6:**
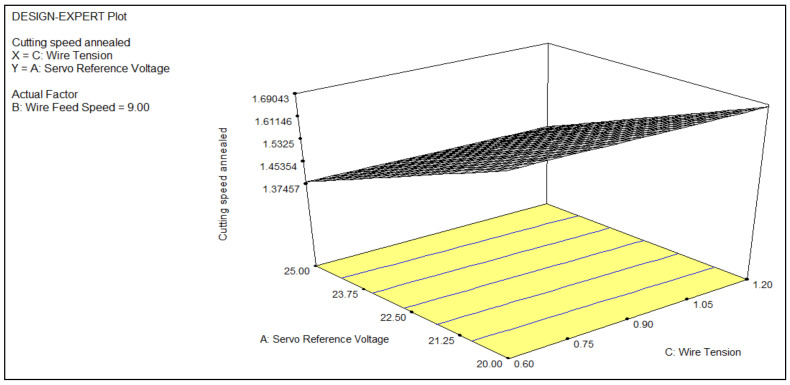
A 3D plot of the cutting speed as a function of the wire tension and servo reference voltage.

Annealing is a heat treatment method that increases a material’s ductility, thereby lowering its hardness. The elimination of dislocations from the crystal structure of the annealed material results in an increase in hardness and ductility. The formability of the material is improved using annealing. It can be difficult to bend or press hard on brittle materials without causing harm. Annealing has the effect of removing this risk. An improvement in ductility, fracture resilience, thermal and dimensional stability, and creep resistance is achieved by annealing titanium alloys [25]. Annealing improves properties such as the toughness and ductility of an alloy and reforms the grain structure of an alloy [28]. Figure 6 describes the effects of the wire tension and servo reference voltage on the cutting speed when the annealed titanium alloy was processed using WEDM. The overall impact of both the wire tension and servo reference voltage caused an increase in the cutting speed when the annealed titanium alloy was processed using WEDM. The better properties improved the spark formation, which led to more vaporization and melting of the workpiece, resulting in a higher cutting speed. The equation for the cutting speed when machining the annealed titanium alloy was as follows:

(2) $$\begin{array}{ll} Cutting\;Speed\; & \left(Anneal\right)\\ & =+2.96740-0.045313\times V_{sr}-0.11445\times F_{w}+0.62678\times T_{w}+2.5000E-003\times V_{sr}\times F_{w}\\ & -8.33333E-003\times V_{sr}\times T_{w}-0.020833\times F_{w}\times T_{w}-6.97650E-004\times {V_{sr}}^{2} \end{array}$$

### 3.3. Analysis of the Oil-Quenched Titanium Alloy

**Figure 7:**
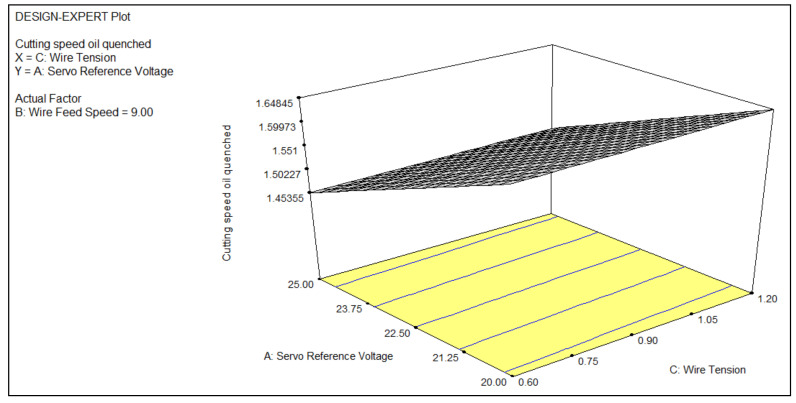
A 3D plot of the effect of the wire tension and servo reference voltage on the cutting speed.

As part of some heat-treatment processes, metals are quenched. Quenching involves rapid cooling of the metal to change the mechanical properties of the initial state. To perform the quenching process, the metal is heated to a temperature higher than that of natural conditions, normally above its recrystallization temperature but below its melting temperature. The immediate cooling of a workpiece in oil (Witmans, Daman, India) to acquire specific material properties is known as oil quenching. Quenching prevents undesirable low-temperature processes, such as a phase transition, from occurring [29,30]. Figure 7 represents the impact of the wire feed and servo reference voltage on the cutting speed while processing the oil-quenched titanium alloy using WEDM. The cutting speed increased with the increase in wire tension and servo voltage. The combined impact of the wire tension and servo reference voltage led to an increase in the cutting speed of the oil-quenched titanium alloy when processed using WEDM. Due to the phase transformation, the titanium alloy became more refined in structure. The grain became reoriented and a more compact structure was obtained. The machining of the oil-quenched titanium alloy resulted in a better cutting speed. The structured orientation of grains resulted in a proper discharge formation, which resulted in more MRR while processing the alloy using WEDM [31]. The equation for the cutting speed when machining the oil-quenched titanium alloy was as follows:

(3) $$\begin{array}{ll} Cutting\;Speed\; & \left(Oil\;Quenched\right)\\ & =+4.11016-0.12227\times V_{sr}-0.15806\times F_{w}+0.43326\times T_{w}+6.5000E-003\times V_{sr}\times F_{w}\\ & -1.6667E-003\times V_{sr}\times T_{w}-0.045833\times F_{w}\times T_{w}+5.60470E-004\times {V_{sr}}^{2} \end{array}$$

### 3.4. Analysis of the Hardened Titanium Alloy

Hardening is an important act in metallurgy that is used to increase metal hardness. The metal hardness is directly proportional to the yield stress at the imposed strain spot. The grain structure becomes refined with the hardening of a titanium alloy [32]. The use of this process results in an enhancement of the mechanical properties, as well as a rise in the degree of toughness, resulting in a tougher, more robust component. Alloys are heated above the critical transition temperature for the substance and are cooled easily enough to turn the fragile original material into a much smoother, stronger structure. The refinement of the grain structure increases the wear resistance of metal and makes the metal tougher. The above plot in Figure 7 represents the effect of two parameters, i.e., the servo reference voltage and wire tension in combination, on the cutting speed of the hardened titanium alloy. The cutting speed increased with the combined increase in the wire tension and servo reference voltage. The hardening of the alloy had a direct impact on the machining parameters while performing manufacturing operations on them [33]. The refinement of the grain structure made the alloy tougher, which led to better surface properties and cutting speed while performing machining operations using the WEDM process [34].

The cutting speed equation for the hardened titanium alloy was as follows:

(4) $$\begin{array}{ll} Cutting\;Speed\; & \left(Hardened\right)\\ & =+5.97863-0.20669\times V_{sr}-0.36113\times F_{w}-0.51005\times T_{w}+0.015000\times V_{sr}\times F_{w}\\ & -0.016667\times V_{sr}\times T_{w}+0.083333\times F_{w}\times T_{w}+1.14253E-003\times {V_{sr}}^{2} \end{array}$$

### 3.5. Surface Characterization

The growth of deep and wide overlapping craters, debris, microcracks, and the recast layer was shown to be significantly influenced by the wire tension, wire feed, and servo reference voltage. During WEDM, the discharge energy encroaches on the workpiece, melting the material and flushing debris away. The surface integrity of a machined component is affected by the thermal action of the EDM process. As a result, the surface integrity analysis was carried out in order to check the parametric effects on the surface defects, such as fractures, voids, and roughness, as well as the thickness of the recast layer deposited on the machined surface.

The nature of a machined surface is mostly described in terms of the surface geography, which incorporates miniaturized scale breaks, a heat-influenced zone, a recast layer, and stage changes in surface and subsurface locales [34]. The surface properties may be changed because of the impact of the procedure parameters, for example, the servo reference voltage, wire tension, and wire feed.

The machined surface had globules of debris, spherical fragments, craters, and micro cracks, according to the SEM micrographs. The formation of deep craters on the machined surface was caused by an increase in the wire feed and wire stress [35]. These deep and covering pits were formed as a result of repeated electrical discharges, which resulted in intense heat being transferred to the specimen’s surface, causing restricted dissolving or evaporation of the work material [36,37]. The deionized water took away a portion of the molten material that was created by the discharge [38,39]. The oxidation reaction, which produces a significant amount of pressure due to a confined spark hole field, is the most likely explanation for deep and massive craters [40,41,42,43,44,45,46].

**Figure 8:**
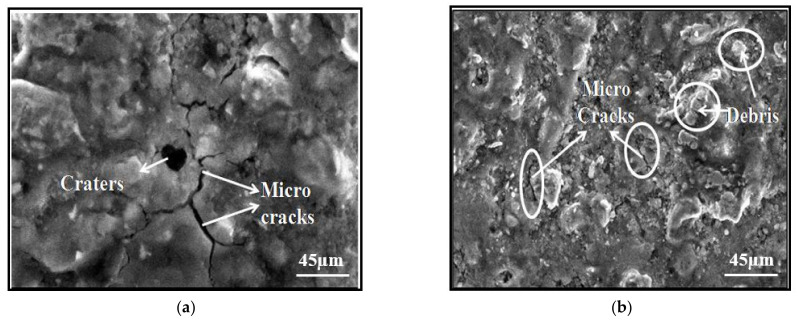

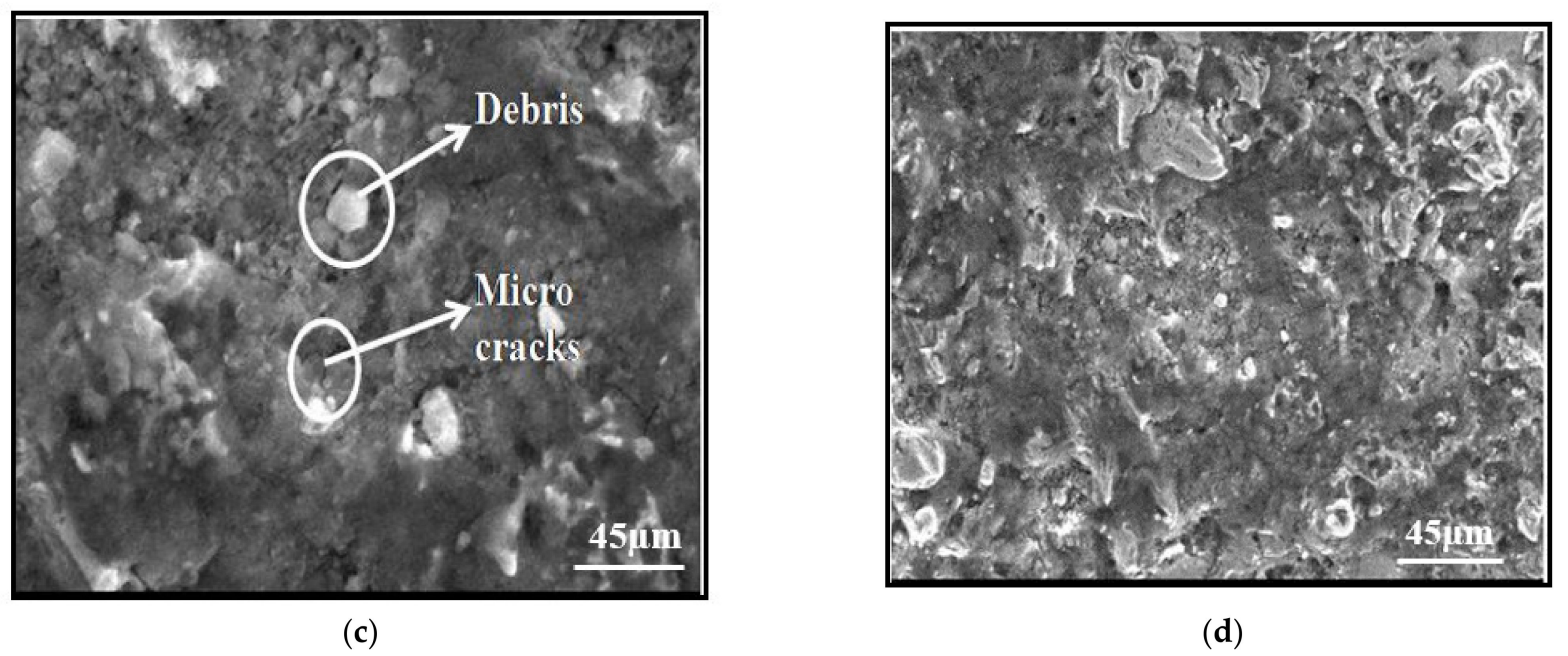
SEM micrographs of the (**a**) pure titanium alloy at 3000×, (**b**) annealed titanium alloy at 1000×, (**c**) oil-quenched titanium alloy at 3000×, and (**d**) hardened titanium alloy at 1000×.

Here, the SEM images of the different processed titanium alloy samples that were machined using WEDM are shown in Figure 8. The differently treated samples were compared in terms of the surface topography, debris formation, and microcrack formation. The SEM images were taken to evaluate how the various process parameter settings affected the surface roughness in various cases. In comparison to other refined alloys, the hardened titanium alloy had stronger surface properties according to the SEM micrographs. Very few cracks and microholes and a small amount of debris formation was observed on the machined surface of the hardened alloy. This was because the hardening of the alloy relieved the internal stresses and the smaller grain formation resulted in a more compact structure. The metal became more refined and hence had better surface properties than the samples that underwent other heat treatments. The lengths of the cracks varied from 4.6 to 20.2 µm.

## 4. Conclusions

The following statements can be made after studying and observing the machining of the titanium alloy using WEDM:

1. The effects of the process parameters, viz. servo reference voltage, wire tension, and wire feed, on the response characteristics, viz. cutting speed, were studied.
2. In the WEDM of the titanium superalloy (Ti-6Al-4V), the RSM was used to build a mathematical model in the form of multiple regression equations that correlated the dependent and independent parameters. The response surfaces were plotted using the model equations to investigate the effects of the process parameters on the performance measures.
3. There was a large increase in the cutting speed when the annealed titanium alloy was processed using WEDM. The increase in cutting speed was greater compared to the other processing methods used on the hardened, quenched, and pure Ti-6Al-4V. The annealing of the titanium alloy increased its ductility at room temperature, fracture toughness, thermal and dimensional stability, and creep resistance. The annealing resulted in the reformation of the microstructures of the titanium alloy, which led to a high cutting speed. The maximum cutting speed of 1.75 mm/min was obtained when the annealed Ti-6Al-4V was processed using WEDM.
4. The Ti-6Al-4V alloy’s properties became refined when it was treated with different heat treatment processes. The alloy became more tough and compact due to changes in the grain structure and the phase transformation.
5. The spark formation was better and more effective in the case of the annealed titanium alloy as compared to the other heat-treated alloy when machined using WEDM.
6. Less breakage of the wire electrode was observed when the annealed alloy was machined using WEDM as compared to the other alloys.
7. The major parameters that impacted the cutting speed the most were the servo reference voltage and the wire tension.
8. The SEM micrographs revealed that the hardened titanium alloy had better surface properties as compared to other processed alloys. There was less formation of microcracks, holes, and debris on the surface of the hardened alloy. This was because of the hardening of the alloy, as it relieved the internal stresses and the smaller grain formation resulted in a more compact structure. The metal became more refined, which allowed for better surface properties than the other alloys.
9. The SEM images revealed that the surface roughness of the hardened alloy was significantly better than the alloys treated with the other heat treatment processes.

## Figures and Tables

**Table 1:** Properties of the Ti-6Al-4V alloy [6].

Properties	Value
Hardness (HRC)	36
Density (g/cc)	4.43
Ultimate tensile strength (MPa)	950
Fatigue strength (MPa)	510
Modulus of elasticity (GPa)	113.8
Elongation (%)	14
Poisson’s ratio	0.342
Thermal conductivity (W/m·K)	6.7
Specific heat (J/kg·K)	560
Electrical resistivity (µΩ·m)	1.78
Melting point (°C)	1604–1660

**Table 2:** Composition of the titanium alloy (Ti-6Al-4V).

Component	Wt.%
Al	≈6
Fe	Max 0.25
O	Max 0.20
Ti	≈90
V	≈4

**Table 3:** Various process parameters range values.

Name	Units	Type	Changes	Standard Deviation	Low	High
Servo reference voltage	V	Factor	Easy	0	18	27
Wire feed speed	m/min	Factor	Easy	0	8	10
Wire tension	N	Factor	Easy	0	0.3	1.4

**Table 4:** Experimental design according to the RSM.

Std	Run	Factors			Cutting Speed (mm/min)			
		A: Servo Reference Voltage (V)	B: Wire Feed Speed (m/min)	C: Wire Tension (N)	Pure Sample	Annealed	Oil Quenched	Hardened
8	1	25	10	1.2	1.50	1.35	1.60	1.55
12	2	22.5	10	0.9	1.45	1.55	1.55	1.50
6	3	25	8	1.2	1.42	1.35	1.40	1.35
7	4	20	10	1.2	1.60	1.7	1.68	1.65
14	5	22.5	9	1.4	1.45	1.50	1.50	1.45
2	6	25	8	0.6	1.40	1.35	1.45	1.40
1	7	20	8	0.6	1.60	1.70	1.60	1.60
5	8	20	8	1.2	1.60	1.70	1.67	1.65
9	9	18.2	9	0.9	1.65	1.75	1.70	1.65
20	10	22.5	9	0.9	1.50	1.50	1.55	1.50
10	11	26.7	9	0.9	1.35	1.30	1.37	1.35
11	12	22.5	7	0.9	1.45	1.55	1.50	1.45
17	13	22.5	9	0.9	1.50	1.60	1.55	1.50
18	14	22.5	9	0.9	1.5	1.6	1.55	1.5
4	15	25	10	0.6	1.5	1.4	1.47	1.45
13	16	22.5	9	0.3	1.55	1.5	1.55	1.55
19	17	22.5	9	0.9	1.5	1.55	1.55	1.5
16	18	22.5	9	0.9	1.5	1.5	1.55	1.5
15	19	22.5	9	0.9	1.45	1.5	1.55	1.5
3	20	20	10	0.6	1.6	1.7	1.68	1.55

## Data Availability

Relevant data is available with corresponding author.
